# The Systemic Inflammome of Severe Obesity before and after Bariatric Surgery

**DOI:** 10.1371/journal.pone.0107859

**Published:** 2014-09-19

**Authors:** Ebymar Arismendi, Eva Rivas, Alvar Agustí, José Ríos, Esther Barreiro, Josep Vidal, Robert Rodriguez-Roisin

**Affiliations:** 1 Servei de Pneumologia (Institut Clínic del Tòrax), Hospital Clínic, Barcelona, Spain; 2 Fundació Clínic per la Recerca Biomèdica, Hospital Clínic, Barcelona, Spain; 3 CIBER Enfermedades Respiratorias (CIBERES), Barcelona and Palma de Mallorca, Spain; 4 Institut d'Investigacions Biomédiques August Pi i Sunyer (IDIBAPS), Universitat de Barcelona, Barcelona, Spain; 5 Servei de Anestesiologia i Reanimació, Hospital Clínic, Barcelona, Spain; 6 Biostatistics and Data Management Core Facility, Biostatistics Unit, Universitat Autònoma de Barcelona, Barcelona, Spain; 7 Pulmonology Department, Hospital del Mar, and Universitat Pompeu Fabra, Parc de Recerca Biomèdica de Barcelona (PRBB), Barcelona, Spain; 8 Servei de Endocrinologia (Institut Clínic de Malalties Digestives i Metabòliques), Hospital Clínic, Barcelona, Spain; University of Rochester Medical Center, United States of America

## Abstract

**Introduction:**

Obesity is associated with low-grade systemic inflammation. The “inflammome” is a network layout of the inflammatory pattern. The systemic inflammome of obesity has not been described as yet. We hypothesized that it can be significantly worsened by smoking and other comorbidities frequently associated with obesity, and ameliorated by bariatric surgery (BS). Besides, whether or not these changes are mirrored in the lungs is unknown, but obesity is often associated with pulmonary inflammation and bronchial hyperresponsiveness.

**Objectives:**

We sought to: *(1)* describe the systemic inflammome of morbid obesity; *(2)* investigate the effects of sex, smoking, sleep apnea syndrome, metabolic syndrome and BS upon this systemic inflammome; and, *(3)* determine their interplay with pulmonary inflammation.

**Methods:**

We studied 129 morbidly obese patients (96 females; age 46±12 years; body mass index [BMI], 46±6 kg/m^2^) before and one year after BS, and 20 healthy, never-smokers, (43±7 years), with normal BMI and spirometry.

**Results:**

Before BS, compared with controls, all obese subjects displayed a strong and coordinated (inflammome) systemic inflammatory response (adiponectin, C-reactive protein, interleukin (IL)-8, IL-10, leptin, soluble tumor necrosis factor-receptor 1(sTNF-R1), and 8-isoprostane). This inflammome was not modified by sex, smoking, or coexistence of obstructive sleep apnea and/or metabolic syndrome. By contrast, it was significantly ameliorated, albeit not completely abolished, after BS. Finally, obese subjects had evidence of pulmonary inflammation (exhaled condensate) that also decreased after BS.

**Conclusions:**

The systemic inflammome of morbid obesity is independent of sex, smoking status and/or comorbidities, it is significantly reduced by BS and mirrored in the lungs.

## Introduction

Obesity is a major and raising global health problem. Among others, it increases significantly the risk of cardiovascular disease and premature death [1]. A key mechanism explaining this association appears to be the release by adipocytes of the so-called adipokines, such as leptin and adiponectin [2], a family of mediators that influences body weight homeostasis, insulin resistance and inflammation, and eventually causes endothelial dysfunction and atherosclerosis [3]. In addition, external risk factors such as smoking often contribute to enhance the adverse effects of obesity on cardiovascular health [3].

Bariatric surgery (BS) results in significant and sustained weight loss in morbidly obese subjects with minor morbidity or mortality [4]–[6]. Previous studies indicate that systemic inflammation in obese subjects appears to be reduced after BS [4]; [7]. Notwithstanding, the inflammatory response is complex and includes the contribution of many different cells and mediators [8]. Network analysis allows a more comprehensive approach to complex biological systems [9] with the potential of unraveling novel interplays among apparently disconnected mediators and clinical manifestations [10]; [11]. This research strategy has already proved to be useful to dissect the biological and environmental determinants of obesity [12] and smoking [13], as well as to characterize the systemic inflammatory pattern (so-called, inflammome) associated with smoking and chronic obstructive pulmonary disease (COPD) [8]. In this study we describe for the first time the *inflammome* of morbid obese individuals and test the hypothesis that it could be significantly worsened by smoking or other comorbidities frequently associated to severe obesity, such as the obstructive sleep apnea syndrome (OSAS) and the metabolic syndrome (MS) and be ameliorated after BS. Besides, given that obesity is often associated with pulmonary inflammation and bronchial hyperresponsiveness [14]–[16], we also sought to investigate potential relationships between systemic and pulmonary inflammation in the morbid obese, so that we also quantified a number of inflammatory markers in the exhaled gas condensate in morbid obese, both before and after BS.

## Methods

For more information see Methods S1.

### Study Design, Participants and Ethics

This was a prospective, observational study in which we enrolled: *(1)* 129 obese individuals (96 females/33 males; age 46±12 years) with a body mass index (BMI) ≥40 kg/m^2^ (or ≥35 kg/m^2^ in those with comorbidities) without major cardiovascular and chronic obstructive airway diseases, candidates to BS; and, *(2)* 20 healthy, normal weighted, sex- (16 females/4 males) and age-matched (43±7 years) non-smokers with normal spirometry, who served as controls. Obese participants were studied before (mean, 8±4 weeks; median, 5 weeks) and one year (15±4 months; median, 13 months) after BS. The project was approved by the Ethics Committee for Clinical Research (*Comitè Ètic d*'*Investigació Clínica*) of Hospital Clínic of Barcelona (2008/4015) and all participants signed their written informed consent.

### Measurements

The following measurements were obtained in all obese subjects before and after BS. Forced spirometry, plethysmographic lung volumes, arterial blood gases and the 6-minute walking test (6MWT) were determined according to international recommendations [17]–[19]. Reference values were those of Roca *et al.* [20]–[22]. An apnea/hypopnea index (AHI) ≥15 events/h was considered indicative of OSAS [23].

Serum was obtained after overnight fasting by peripheral venopuncture followed by centrifugation and stored at −80°C until analysis. The serum concentration of adiponectin, C-reactive protein (CRP), interleukin (IL)-8, IL-10, leptin, soluble tumor necrosis factor-receptor 1(sTNF-R1), and 8-isoprostane were determined, as previously reported [24]. The serum concentration of C-reactive protein (CRP) were determined using an immunoturbidimetry method (Advia Chemistry, Siemens Tarrytown, NY, USA) and those of leptin (Diagnostic Biochem Canada Inc. Ontario, Canada), serum adiponectin, soluble tumor necrosis factor-receptor 1(sTNF-R1), interleukin (IL)-8, IL-10 and 8-isoprostane by ELISA (US Biological Salem, MA, USA; IBL international Hamburg, Germany; ANOGEN Ontario, Canada and Cayman Chemical Company, Ann Arbor, MI, US, respectively). All biomarkers were quantified in duplicate and their mean values were used for analysis. In some individuals serum biomarker concentrations were below the lower limit of quantification (LLQ). To avoid a downward bias of biomarkers, a nominal level of half of the LLQ value was used in the analysis in individuals with values below the LLQ [25]. Exhaled breath condensate samples were obtained using an EcoScreen condenser (Jaeger, Würzburg, Germany) following international recommendations [26]; [27] and the concentrations of IL-8, IL-10 and 8-isoprostane were measured by ELISA (Cayman Chemical Company, Ann Arbor, MI, US).

Forced spirometry and serum and exhaled biomarker concentrations were determined in control participants only once.

### Statistical Analysis

Results are described as mean ± standard deviation (SD), median [interquartile range] [IQR] or absolute and relative frequencies (%), as appropriate. Quantitative variables, were tested for normality using a Kolgomorov-Smirnov test and parametric (paired and unpaired t-test) and non-parametric (Wilcoxon and Mann-Whitney tests) were used accordingly to compare quantitative variables between patients and controls (at baseline) and between patients before and after BS. Fisher's exact test and McNemar test were used for qualitative variables.

As described previously [8], we used the 95^th^ (and 5^th^) percentile value determined in controls as the upper (and lower) normal levels, so biomarker concentrations beyond these threshold were considered abnormal in obese subjects. Cross tabulations between healthy and obese subjects, before and after BS and also in different subsets of obese individuals according to sex, smoking status and coexistence of OSAS and MS, were determined to analyze biomarker alterations and their interactions. All statistical tests were two-sided and a p value <0.05 was considered significant. Due to the observational characteristics of this study p values presented were nominal and not adjusted for multiplicity. Data analysis was carried out with SPSS 20.0 (IBM Corporation).

## Results

### Characterization of Participants

Table 1 presents the main demographic and clinical characteristics of participants. BMI, waist and waist-to-hip ratio were, as compared to control, higher in obese subjects, but age and proportion of females were similar in the two populations. Only 21 obese subjects were current smokers (≥10 pack-years). Most obese subjects were non- (<10 pack-years) or former (>1 year after cessation) smokers, and their level of dyspnea was mild-to-moderate. Comorbidities were common in obese individuals, especially OSAS (67%) and MS (78%). As shown in Table 2 obese, as compared to control participants, had reduced forced spirometric and pulseoximetry values, although within normal limits, along with diminished expiratory reserve volume (ERV) and functional residual capacity (FRC) values. Mean PaO_2_ (range, 57-119 mmHg) was within normal limits and mean alveolar-arterial PO_2_ difference (AaPO_2_) was abnormally enlarged (range, 0–51 mmHg). The former two values were more abnormal in males than in females (Tables S1 and S2).

**Table 1:** Main demographic and clinical characteristics of control and obese participants (mean ± SD or n (%)).

	CONTROL SUBJECTS		OBESE SUBJECTS		
		*P* Value *	BEFORE BS	*P* Value †	AFTER BS
**Demographics**					
Age, years	43±7	0.7	46±12	0.68	47±12
Female,%	83	0.24	74	---	74
Body mass index, kg/m^2^	22±3	<.001	46±6	<.001	30±5
Waist circumference, cm	80±8	<.001	130±14	<.001	99±13
Waist-to-hip ratio	0.84±0.09	0.001	0.93±0.09	0.016	0.89±0.09
**Clinical features**					
Non-Smokers, n (%)	20	<.001	75 (58)	<.001	75 (58)
Current smokers, n (%)	0	<.001	21 (16)	.08	17 (13)
Tobacco, pack-years	0	<.001	34±32	.36	35±32
Ex-smokers, n (%)	0	<.001	33 (26)	.43	37 (29)
Tobacco, pack-years	0	<.001	35±24	.97	35±24
Dyspnea level (mMRC)	0	<.001	1.2±0.8	<.001	0.1±0.3
Obstructive Sleep Apnea, n (%)	—	NA	87 (67)	<.001	13 (10)
Apnea Hypopnea Index, events/h	—	NA	60±34	<.001	17±15
Metabolic Syndrome, n (%)	0	<.001	100 (78)	<.001	20 (16)
Diabetes Mellitus type 2, n (%)	0	<.001	52 (40)	<.001	12 (9)
Hypertension, n (%)	0	<.001	77 (60)	<.001	37 (29)

Demographic and clinical characteristics of healthy and obese individuals, before and after bariatric surgery. NA: not applicable; * p-values for comparisons between controls individuals and obese subjects before bariatric surgery whereas † indicate p-values for comparisons between obese subjects before and after bariatric surgery.

**Table 2:** Lung function and inflammatory markers in control and obese participants, before and after bariatric surgery (mean ± SD or median [interquartile range]).

	CONTROL SUBJECTS		OBESE PATIENS		
		*P* Value *	BEFORE BS	*P* Value †	AFTER BS
**Lung function**					
FVC,% pred	103±13	0.003	91±13	<.001	103±13
FEV_1_,% pred	102±13	0.02	94±15	<.001	104±14
FEV_1_/FVC,%	71±4	0.008	82±5	<.001	79±9
FRC,% pred	ND	---	73±13	<.001	113±25
ERV,% pred	ND	---	34±23	<.001	106±36
TLC,% pred	ND	---	92±10	<.001	106±13
RV/TLC,%	ND	---	35±7	0.14	36±8
SGaw, s-1·cmH2O-1	ND	---	0.11±0.04	0.002	0.13±0.10
PaO_2_, mmHg	ND	---	82±12	<.001	93±11
PaCO_2_, mmHg	ND	---	37±4	<.001	39±5
AaPO_2_, mmHg	ND	---	23±10	<.001	9±11
SaO_2_,%	98±1	0.046	97±3	0.36	97±7
6MWT, m	ND	---	471±75	<.001	546±76
**Serum markers**					
Leucocytes, 10^9^/l	6,215 [5490–7682]	<.001	8,010 [6,825–9,395]	<.001	6,700 [5,600–7,855]
C-Reactive Protein, mg/l	0.40 [0.16–0.70]	<.001	7.80 [4.30–14.50]	<.001	0.60 [0.20–1.45]
Fibrinogen, mg/dl	320 [280–350]	<.001	420 [368–480]	<.001	370 [333–438]
Leptin, ng/ml	13.60 [5.66–18.93]	<.001	63.00 [42.85–101.35]	<.001	15.00 [6.70–28.85]
Adiponectin, µg/ml	22.66 [18.70–25.92]	<.001	9.58 [4.88–15.85]	<.001	17.12 [9.68–22.55]
sTNF-R1, ng/ml	0.24 [0.07–0.43]	<.001	1.50 [1.01–2.24]	<.001	0.89 [0.34–1.61]
IL-8, pg/ml	4.00 [4.00–5.56]	0.019	9.22 [4.00–25.02]	0.012	4.00 [0.98–13.66]
IL-10, pg/ml	3.50 [3.50–148.54]	0.72	3.50 [3.50–16.59]	0.46	3.50 [3.50–11.45]
8-isoprostane, pg/ml	40.24 [23.90–58.50]	<.001	162.25 [108.30–211.90]	0.26	163.30 [93.27–213.36]
**Exhaled condensate markers**					
Exhaled IL-8, pg/ml	0.60 [0.33–1.36]	<.001	4.77 [2.21–8.74]	0.019	3.83 [1.26–6.79]
Exhaled IL-10, pg/ml	4.71 [2.32–7.46]	<.013	8.84 [4.75–15.24]	0.004	6.13 [4.07–10.29]
Exhaled 8-isoprostane, pg/ml	350.91 [177.31–603.23]	0.018	231.80 [113.15–362.69]	0.86	216.00 [129.65–372.65]

ND: Not done; FRC: functional residual capacity; ERV: expiratory reserve volume; TLC: total lung capacity; RV: residual volume; SG_aw_: specific conductance; 6MWT: 6-minute walking test; sTNF-R1: soluble tumor necrosis factor-receptor 1; IL: interleukin. * p-values for comparisons between controls individuals and obese subjects before bariatric surgery whereas † indicate p-values for comparisons between obese subjects before and after bariatric surgery.

### Systemic Inflammation

**Figure 1:**
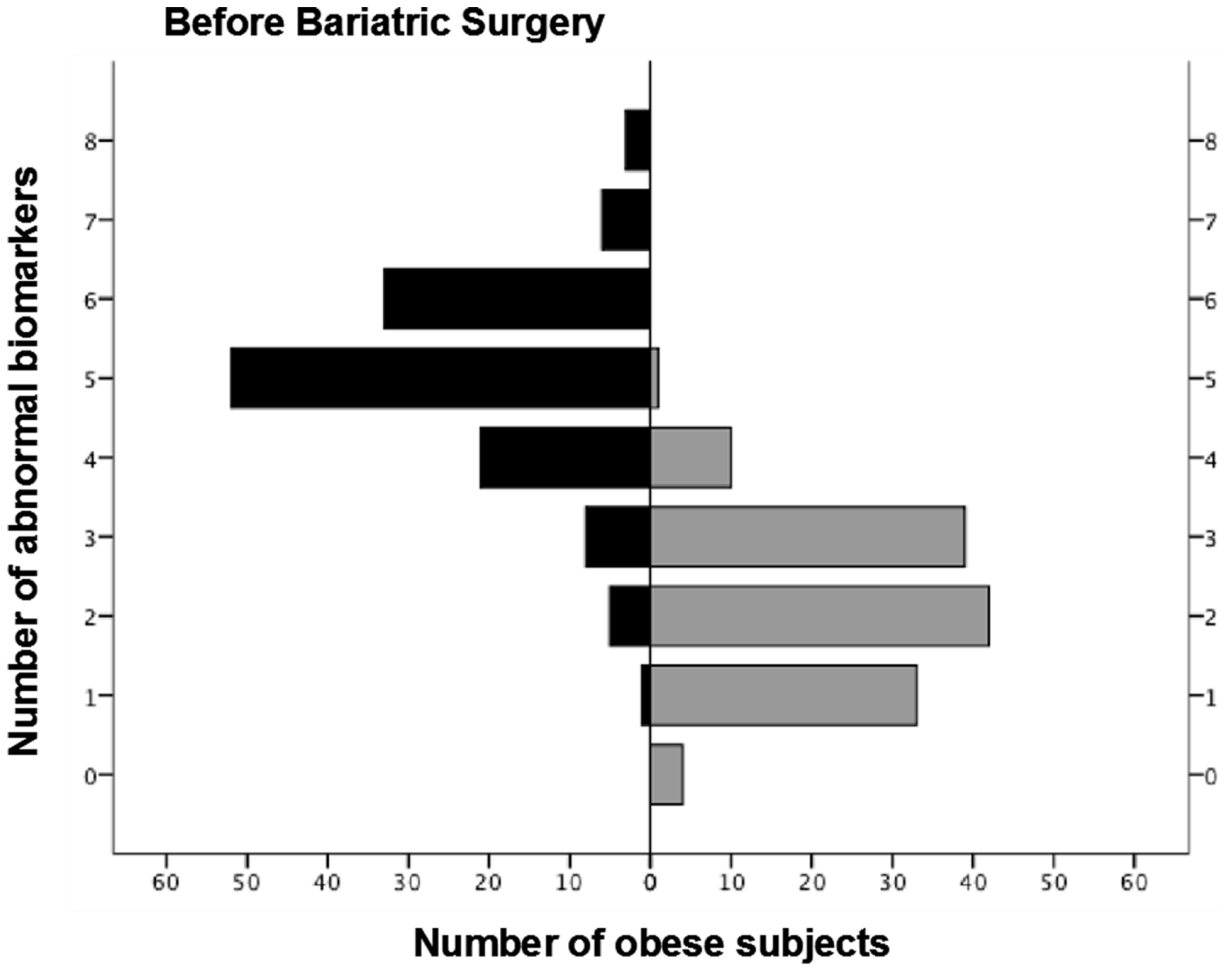
Frequency distribution of obese individuals according to the number of abnormal systemic (serum) biomarker values (>95^th^ percentile of controls (or <5^th^ percentile in the case of adiponectin), before and after BS.

The mean concentration of most serum inflammatory biomarkers was significantly higher in obese than in control subjects, except for adiponectin, which was lower (Table 2). Figure 1 presents the frequency distribution of the number of abnormal serum biomarker values in obese individuals (>95^th^ percentile of controls (or <5^th^ percentile in the case of adiponectin) [8]. Not a single obese subject had a normal battery of biomarkers and the majority exhibited at least 5 or more abnormal biomarkers. We observed that BMI was significantly associated to the serum concentrations of CRP (Rho, 0.31; p<0.001) and leptin (Rho, 0.41; p<0.001) values, whereas descriptors of central adiposity, *i.e.* waist circumference, were associated to sTNF-R1 (Rho, 0.25; p<0.01) and adiponectin (Rho, -0.18; p<0.05) levels.

**Figure 2:**
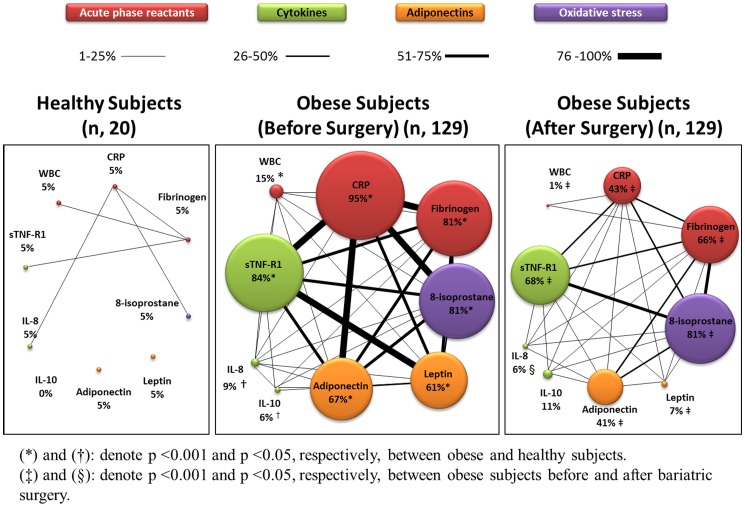
Systemic inflammome in healthy and obese individuals before and after BS. Each node represents one inflammatory marker and color indicates the type of inflammatory marker considered (acute phase reactants, cytokines, adipokines or oxidative stress). The node diameter is proportional to the prevalence of abnormal values (i.e.,>95^th^ or <5^th^ of controls) of that particular biomarker in the population under consideration (control or obese individuals) and the thickness of the edges linking pairs of nodes is proportional to the prevalence of co-occurrence of abnormal biomarkers of that particular pair of nodes.

The systemic inflammome is a network representation (Figure 2) of the prevalence and relationships between the different inflammatory markers determined in serum [8]; [28]. In obese subjects, all nodes were significantly larger than in control participants (indicating a higher prevalence of abnormal values) and there were many significant interactions among the different inflammatory biomarkers determined (Figure 2). By contrast, in control subjects, nodes were by definition small, many of them were not linked to the others and, in any case, links were few and thin, indicating the virtual absence of systemic inflammation.

We explored the effects of a number of factors that can potentially influence the systemic inflammome of morbid obesity, including sex, smoking status, and coexistence of OSAS or MS [24]; [29]–[31]. By and large, differences between males and females (Figure S1), current or former and non-smokers (Figure S2), and/or participants with or without OSAS and/or MS (Figures S3 and S4, respectively) were modest or absent, indicating that morbid obesity by itself was the main driving force of the systemic inflammome in these patients. Nevertheless, some (small) changes deserve comment.

### Pulmonary Inflammation

A large proportion of obese subjects had abnormal levels (>95^th^ percentile of controls) of exhaled IL-8 (56%) and IL-10 (15%) so that their mean values were significantly higher in obese subjects (Table 2). On the other hand, albeit exhaled 8-isoprostane was within the normal range in all obese individuals, mean values were significantly lower (Table 2). We did not observe significant differences in any of these exhaled inflammatory markers according to sex, smoking status and/or presence of OSAS and/or MS. The AHI and the concentration of exhaled breath biomarkers were not significantly related. By contrast, we observed a positive correlation between the concentration of exhaled IL-8 and serum sTNF-R1 (Rho, 0.24; p<0.01) and between exhaled IL-8 and serum 8-isoprostane (Rho, 0.27; p<0.01) as well.

### Findings One Year after Bariatric Surgery

Sleeve gastrectomy was performed in 68 (53%) and Roux-en-Y gastric bypass in 61 (47%) obese subjects. Ninety-one percent of obese subjects (n = 118) had an excess weight loss>50% (75±18%), a marker of BS success [4].

Tables 1 and 2 show that most clinical and functional outcomes improved and most inflammatory markers were reduced after BS. All lung function tests improved significantly after BS but it should be noted that they were already within normal limits before surgery. It is of note that the 6MWT substantially increased and this was a novel post-operative finding in morbid obese.

The effects of BS upon systemic inflammation are illustrated in Figures 1 and 2. There was a dramatic downward shift of the frequency distribution of obese subjects with abnormal biomarkers with a marked shrinking of the systemic inflammome after BS, both in terms of node size and link width. Of note, however, that some patients still remained inflamed after BS (Figures 1 and 2). Finally, we observed that the exhaled concentrations of IL-8 and IL-10 (but not those of 8-isoprostane) were also significantly reduced after BS (Table 2).

## Discussion

This study describes, for the first time to our knowledge, the systemic inflammome associated with morbid obesity and shows that it is: *(1)* barely modified by sex, smoking status and/or coexistence of OSAS and/or MS; *(2)* significantly reduced, albeit not fully normalized, after BS; and, *(3)* related to pulmonary inflammation.

### Previous Studies

Several previous studies have shown that severe obesity is associated with systemic inflammation that is considerably reduced after BS [4]; [7]. Our findings confirm and expand these previous results by providing an integrated network approach of the interplay among the different inflammatory markers (inflammome) as well as the effects of potential confounders, such as sex, smoking, OSAS and/or MS, and BS. This approach has been used successfully in other diseases, such as COPD [8]; [32]. On the other hand, it is also worth noting that many previous papers have also investigated the effects of obesity on lung function [14]–[16];[33], including a recent report by our group that used the multiple inert gases elimination technique (MIGET) to investigate the pulmonary and non-pulmonary factors governing gas exchange in a small subset of females [24].

### Interpretation of Findings

Several observations of our study deserve specific discussion. First, our results confirm [3]; [34]–[35] that morbid obesity is associated with a notable systemic inflammation component (Figure 1), here illustrated for the first time as an inflammome (Figure 2). Adipose tissue is an active endocrine organ capable of producing cytokines and hormones that regulate metabolism and immune responses [36]. Hotamisligil *et al.* coined the term “*meta-inflammation*” (metabolically triggered inflammation) to describe a condition triggered by nutrients that engages a similar set of molecules and signaling pathways to those involved in other, more classical, forms of inflammation [37]. It is also known that, in obesity, the hypertrophic adipose tissue becomes infiltrated with pro-inflammatory macrophages, produces more pro-inflammatory cytokines and less adiponectin (an anti-inflammatory adipokine) and contributes to the systemic complications of obesity, including diabetes type 2 and MS, as well as to increased cardiovascular risk in these populations [37]–[38].

Second, contrary to our working hypothesis, we were not able to identify a clear effect of sex, smoking, OSAS or MS upon the systemic inflammome of morbidly obese individuals (Figures S1–S4), indicating that obesity *per se* is likely the main driving force of systemic inflammation in this clinical setting. By contrast, we observed a very significant effect of BS (Figure 2). Our findings confirm that BS is a safe and effective option for the treatment of very severe obesity but also showed that it has a profound effect on the systemic inflammome of these individuals (Figures 1 and 2). This may be related to the reduction of macrophage infiltration of adipose tissue, as well as to the change in the pro-inflammatory macrophage phenotype that has been reported after weight loss [39]. This further supports a key role of obesity in the pathobiology of systemic inflammation in these patients.

Finally, an important novel observation of our study relates to pulmonary inflammation in morbid obesity. In keeping with previous studies [30]–[31]; [40], we found evidence of airway inflammation in obese subjects (Table 2), but our results extend and complement these previous reports by showing that there was a significant interplay between systemic and pulmonary biomarkers and, notably, that BS not only reduced systemic inflammation but had a similar anti-inflammatory effect in the lungs as well. Most previous studies of pulmonary inflammation in obese subjects included individuals with OSAS [30]–[31]; [42], which was in fact believed to be the main pathogenic driver of the observed pulmonary inflammation. In contrast, we observed that airway inflammation was not different in obese patients with or without OSAS (and/or MS, or smoking). In keeping with these observations, a recent study in adults with obesity and OSAS has shown that the combined use of CPAP and weight-loss did not reduce serum CRP levels more than either intervention alone [43]. The fact that pulmonary inflammation was significantly reduced after BS in our study further supports the key role played by obesity in the pathogenesis of both systemic and pulmonary inflammation. In closing, exhaled 8-isoprostane, derived from free radical-catalyzed peroxidation of arachidonic acid, is a reliable biomarker of oxidative stress [44]–[45]. Pre-operative exhaled breath condensate levels of 8-isoprostane in obese patients were lower than in control participants (Table 2), suggesting that either oxidative stress does not play a key role in airway inflammation of morbidly obese subjects and/or that these individuals have developed a more efficient anti-oxidant capacity.

### Strengths and Limitations

Our study has both strengths and limitations. As alluded to this is the first study to use a more comprehensive network approach to investigate the inflammatory pattern associated with morbid obesity, as well as the effects of potential confounding factors and BS, both in the systemic and pulmonary compartments. We acknowledge that we quantified a relatively low number of biomarkers and that we did not measure their levels in adipose or lung tissue.

## Conclusion

Morbid obesity is associated with a significant systemic inflammome that is not influenced by sex, smoking status, presence of obstructive sleep apnea and/or metabolic syndrome, is related to pulmonary inflammation, and is significantly ameliorated after bariatric surgery.

## Supporting Information

Figure S1 **Systemic inflammome in obese participants classified according to sex before BS (for further explanation, see legend to** **Figure 2** **).**
(TIF)

Figure S2 **Systemic inflammome in obese participants classified according to smoking habits before BS.** Current smokers (≥10 pack-years); non- (<10 pack-years) or former (>1 year after cessation) smokers (for further explanation, see legend to Figure 2).
(TIF)

Figure S3 **Systemic inflammome in obese participants classified according to the presence or absence of obstructive sleep apnea syndrome (OSAS) before BS.** OSAS was define as apnea/hypopnea index>15 events/hour (for further explanation, see legend of Figure 2).
(TIF)

Figure S4 **Systemic inflammome in obese participants classified according to the presence or absence of metabolic syndrome (MS) before BS (for further explanation, see legend to** **Figure 2** **).**
(TIF)

Table S1 **Demographic and clinical characteristics (mean ± SD or median [interquartile range]).**
(DOC)

Table S2 **Functional characteristics and serum and Exhaled Breath Condensate Biomarkers in Obese Individuals divided according to sex.**
(DOC)

Methods S1 Additional information regarding methods.
(DOC)
