# Supplementary figures and images for: The Systemic Inflammome of Severe Obesity before and after Bariatric Surgery
Source: PLoS One. 2014 Sep 19;9(9):e107859. doi: 10.1371/journal.pone.0107859 (PMC4169608; doi:10.1371/journal.pone.0107859)

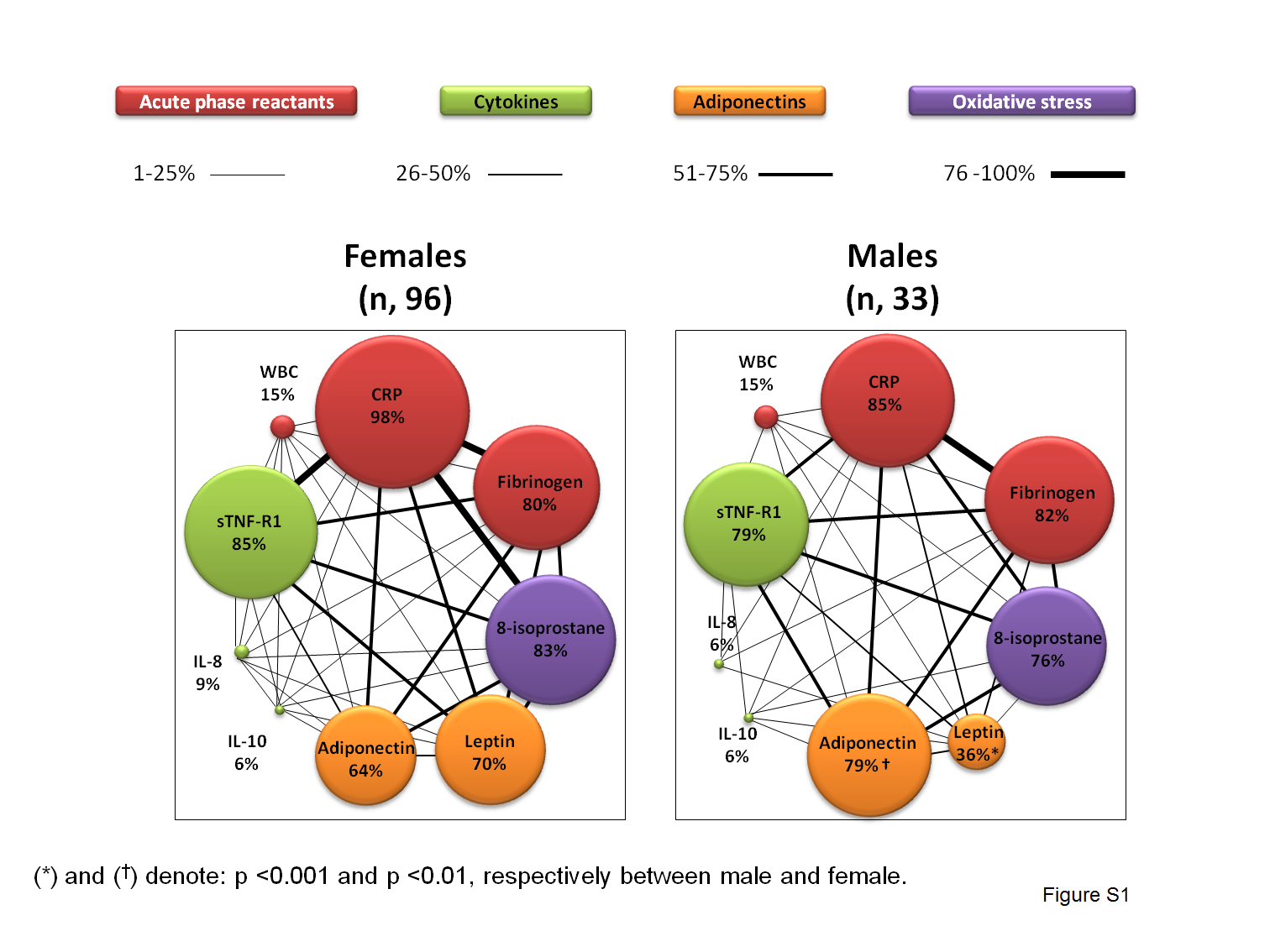

Supplement: Figure S1 — Systemic inflammome in obese participants classified according to sex before BS (for further explanation, see legend to Figure 2 ). (TIF) [file pone.0107859.s001.tif]

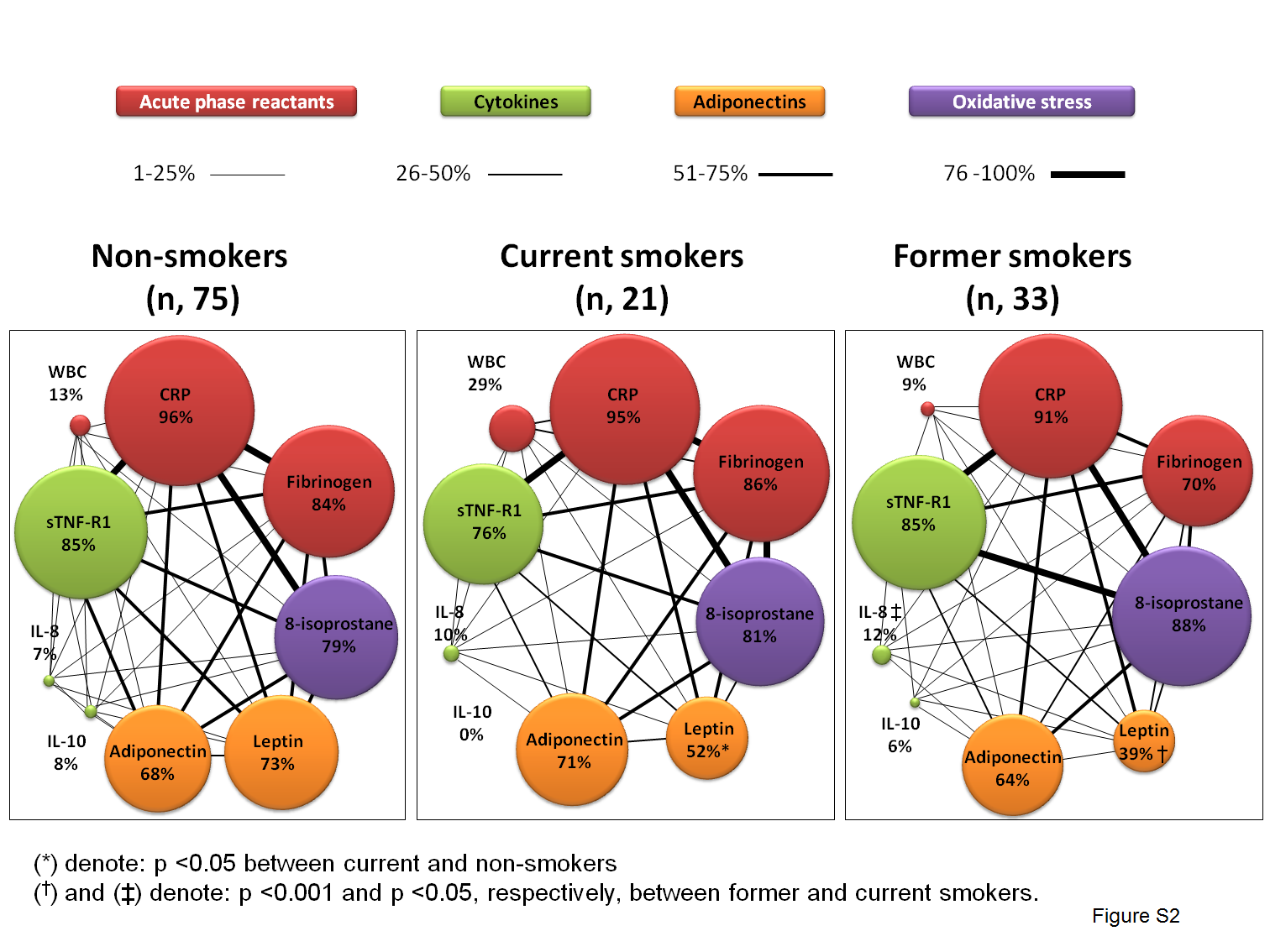

Supplement: Figure S2 — Systemic inflammome in obese participants classified according to smoking habits before BS. Current smokers (≥10 pack-years); non- (<10 pack-years) or former (>1 year after cessation) smokers (for further explanation, see legend to Figure 2). (TIF) [file pone.0107859.s002.tif]

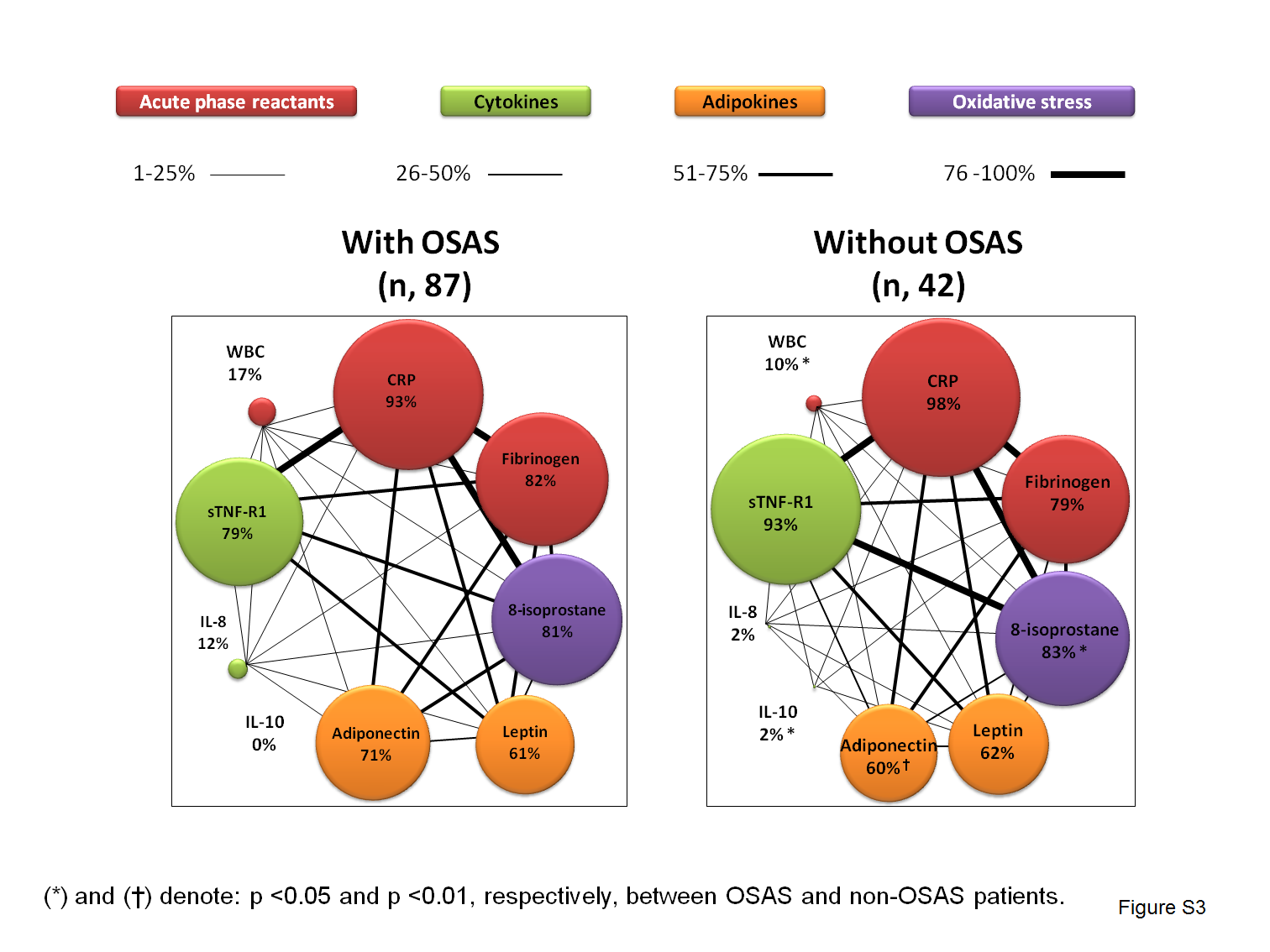

Supplement: Figure S3 — Systemic inflammome in obese participants classified according to the presence or absence of obstructive sleep apnea syndrome (OSAS) before BS. OSAS was define as apnea/hypopnea index>15 events/hour (for further explanation, see legend of Figure 2). (TIF) [file pone.0107859.s003.tif]

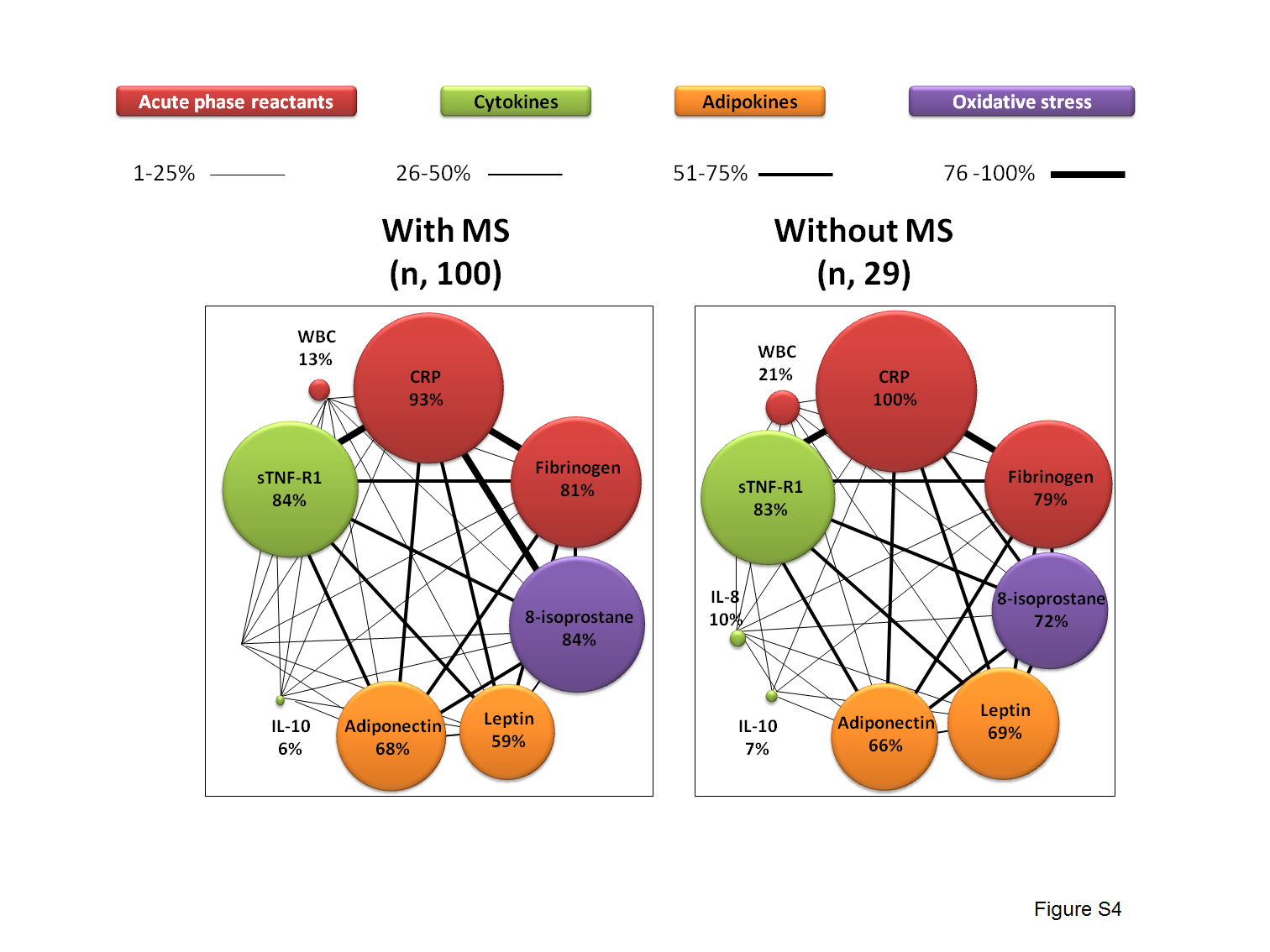

Supplement: Figure S4 — Systemic inflammome in obese participants classified according to the presence or absence of metabolic syndrome (MS) before BS (for further explanation, see legend to Figure 2 ). (TIF) [file pone.0107859.s004.tif]
