# Supplementary material for: The Systemic Inflammome of Severe Obesity before and after Bariatric Surgery
Source: PLoS One. 2014 Sep 19;9(9):e107859. doi: 10.1371/journal.pone.0107859 (PMC4169608; doi:10.1371/journal.pone.0107859)
Supplement: Table S1 — Demographic and clinical characteristics (mean ± SD or median [interquartile range]). (DOC) [file pone.0107859.s005.doc]

| **Table S1**. Demographic and clinical characteristics (mean ± SD or median [interquartile range]). | **BEFORE** | | | **AFTER** | |  |
| --- | --- | --- | --- | --- | --- | --- |
|  | **FEMALES**  **(n, 96)** | **MALES**  **(n, 33)** | ***P* Value ^*^** | **FEMALES**  **(n, 96)** | **MALES**  **(n, 33)** | ***P* Value ^†^** |
| **Demographics** |  |  |  |  |  |  |
| Age, years | 45±12 | 47±11 | .55 | 46±12 | 48±11 | .57 |
| Body mass index, kg/m**^2^** | 46±6 | 46±7 | .46 | 30±4 | 32±5 | .011 |
| Waist circumference, cm | 126±11 | 140±15 | <.001 | 96±11 | 111±13 | <.001 |
| Waist-to-hip ratio | 0.90±0.07 | 1.01±0.08 | <.001 | 0.86±0.08 | 0.96±0.07 | <.001 |
| **Clinical features** |  |  |  |  |  |  |
| Non-Smokers, n (%) | 63 (66) | 12 (36) | .003 | 63 (66) | 12 (36) | .003 |
| Current smokers, n (%) | 14 (15) | 7 (21) | .08 | 11 (12) | 6 (18) | .08 |
| Tobacco, pack-years | 29±18 | 45±51 | .30 | 30±18 | 45±51 | .36 |
| Ex-smokers, n (%) | 19 (20) | 14 (42) | .35 | 22 (23) | 15 (46) | .43 |
| Tobacco, pack-years | 36±25 | 34±25 | .85 | 36±24 | 34±25 | .97 |
| Dyspnea (mMRC) | 1.2±0.7 | 1.2±0.9 | .67 | 0.3±0.6 | 0.3±0.7 | .75 |
| Obstructive Sleep Apnea, n (%) | 54 (56) | 33 (100) | <.001 | 7 (7) | 6 (18) | .08 |
| Apnea Hypopnea Index, events/h | 51±29 | 70±38 | .039 | 14±13 | 24±17 | .024 |
| Metabolic Syndrome, n (%) | 71 (74) | 29 (88) | .08 | 13 (14) | 7 (21) | .22 |
| Diabetes Mellitus type 2, n (%) | 38 (40) | 14 (42) | .47 | 8 (8) | 4 (12) | .37 |
| Hypertension, n (%) | 52 (54) | 25 (76) | .022 | 24 (25) | 13 (39) | .09 |

*p-values for comparisons between females and males before bariatric surgery whereas † indicate p-values for comparisons between females and males after bariatric surgery.
