# Supplementary material for: The Systemic Inflammome of Severe Obesity before and after Bariatric Surgery
Source: PLoS One. 2014 Sep 19;9(9):e107859. doi: 10.1371/journal.pone.0107859 (PMC4169608; doi:10.1371/journal.pone.0107859)
Supplement: Table S2 — Functional characteristics and serum and Exhaled Breath Condensate Biomarkers in Obese Individuals divided according to sex. (DOC) [file pone.0107859.s006.doc]

| **Table S2**. Functional characteristics and serum and Exhaled Breath Condensate Biomarkers in Obese Individuals divided according to sex | **BEFORE** | | | **AFTER** | | |
| --- | --- | --- | --- | --- | --- | --- |
|  | **FEMALES (n, 96)** | **MALES (n,33)** | ***P* Value ^*^** | **FEMALES (n, 96)** | **MALES (n,33)** | ***P* Value ^†^** |
| **Lung function** |  |  |  |  |  |  |
| FVC, % pred | 93±13 | 85±12 | .55 | 104±13 | 100±12 | .09 |
| FEV_1_, % pred | 97±14 | 86±15 | .002 | 105±14 | 98±14 | .013 |
| FEV_1_/FVC, % | 83±5 | 80±7 | <.001 | 80±5 | 74±15 | .044 |
| FRC, % pred | 73±12 | 74±16 | .048 | 113±25 | 112±24 | .90 |
| ERV, % pred | 34±22 | 36±26 | .56 | 103±35 | 114±40 | .14 |
| TLC, % pred | 93±11 | 90±10 | .72 | 105±17 | 104±12 | .74 |
| RV/TLC, % | 35±7 | 34±8 | .32 | 37±7 | 32±7 | .006 |
| SG_aw_, s^-1^·cmH_2_O^-1^ | 0.11±0.03 | 0.09±0.04 | .41 | 0.15±0.11 | 0.10±0.06 | .04 |
| PaO_2_, mmHg | 85±12 | 77±10 | .033 | 94±10 | 91±15 | .22 |
| PaCO_2_, mmHg | 36±4 | 38±3 | .001 | 39±4 | 39±8 | .93 |
| AaPO_2_, mmHg | 22±10 | 27±9 | .010 | 9±9 | 11±15 | .35 |
| SaO_2_, % | 97±1 | 95±6 | .006 | 98±1 | 95±3 | .24 |
| 6MWT, m | 459±69 | 504±82 | .06 | 529±71 | 596±69 | <.001 |
| **Serum markers** |  |  |  |  |  |  |
| Leucocytes, 10^9^/l | 7,895 [6,763-9,200] | 8,200 [6,900-9,985] | .45 | 6,395 [5,522-7,500] | 7,550 [6,010-8,805] | .012 |
| C-Reactive Protein, mg/l | 7.95 [4.60-14.60] | 6.70 [1.45-13.60] | .16 | 0.55 [0.20-1.38] | 0.80 [0.25-1.75] | .63 |
| Fibrinogen, mg/dl | 440 [368-500] | 400 [363-445] | .07 | 3.80 [3.40-4.48] | 350 [320-378] | .019 |
| Leptin, ng/ml | 87.95 [46.03-103.00] | 44.00 [30.35-64.75] | <.001 | 17.10 (9.65-30.40] | 6.90 [2.45-20.80] | .002 |
| Adiponectin, µg/ml | 11.02 [5.51-17.79] | 6.64 [2.79-11.91] | .005 | 18.37 [10.22-24.25] | 14.07 [7.02-18.51] | .017 |
| sTNF-R1, ng/ml | 1.51 [1.02-2.19] | 1.40 [0.61-2.63] | .96 | 0.89 [0.34-1.42] | 0.91 [0.34-1.85] | .52 |
| IL-8, pg/ml | 9.25 [4.00-30.60] | 8.00 [4.00-18.15] | .71 | 4.00 [0.72-12.09] | 6.55 [4.00-46.67] | .035 |
| IL-10, pg/ml | 3.50 [3.50-15.87] | 3.50 [3.50-39.98] | .61 | 3.50 [3.50-4.13] | 3.50 [3.50-32.53] | .20 |
| 8-isoprostane, pg/ml | 172.55 [113.53-213.09] | 144.20 [86.41-221.98] | .23 | 169.52 [107.46-208.21] | 122.30 [50.23-217.30] | .06 |
| **Exhaled condensate markers** |  |  |  |  |  |  |
| Exhaled IL-8, pg/ml | 5.21 [2.71-8.97] | 4.10 [1.53-7.43] | .36 | 3.68 [1.26-6.11] | 4.64 [0.85-9.43] | .35 |
| Exhaled IL-10, pg/ml | 9.52 [5.04-15.52] | 7.15 [3.97-14.77] | .24 | 6.37 [4.07-10.10] | 5.95 [4.08-12.06] | .89 |
| Exhaled 8-isoprostane, pg/ml | 235.91[123.75-345.91] | 205.10 [61.93-448.95] | .57 | 219.55 [146.30-378.75] | 216 [56.70-357.80] | .39 |

Unless otherwise stated, data are expressed as mean ± SD and median (interquartile range); pred: predicted; FRC: functional residual capacity; ERV: expiratory reserve volume; TLC: total lung capacity; RV: residual volume; SG_aw_: specific conductance; 6MWT: 6**-**minute walking test; sTNF-R1: soluble tumor necrosis factor-receptor 1; IL: interleukin; (*****): p-values for comparisons between females and males before bariatric surgery whereas ^†^ for comparison between females and males after bariatric surgery.
