# Supplementary material for: The Systemic Inflammome of Severe Obesity before and after Bariatric Surgery
Source: PLoS One. 2014 Sep 19;9(9):e107859. doi: 10.1371/journal.pone.0107859 (PMC4169608; doi:10.1371/journal.pone.0107859)
Supplement: Methods S1 — Additional information regarding methods. (DOC) [file pone.0107859.s007.doc]

Saturday, August 29, 2026

**SUPPORTING INFORMATION DOCUMENT**

**The Systemic Inflammome of Severe Obesity Before and After Bariatric Surgery**

Ebymar Arismendi MD ^1,2,3,4^, Eva Rivas MD ^4,5^, Alvar Agustí MD ^1,2,3,4^, José Ríos MsC ^4,6^, Esther Barreiro MD, PhD ^3,7^, Josep Vidal MD ^2,4,8^, Robert Rodriguez-Roisin MD ^1,2,3,4^

(1) Servei de Pneumologia (Institut Clínic del Tòrax), Hospital Clínic, Barcelona, Spain; (2) Fundació Clínic per la Recerca Biomèdica, Hospital Clínic, Barcelona, Spain; (3) CIBER Enfermedades Respiratorias (CIBERES) Barcelona and Palma de Mallorca, Spain; (4) Institut d’Investigacions Biomèdiques August Pi i Sunyer (IDIBAPS), Universitat de Barcelona, Barcelona, Spain; (5) Servei de Anestesiologia i Reanimació, Hospital Clínic, Barcelona, Spain; (6) Biostatistics and Data Management Core Facility, Biostatistics Unit, Universitat Autònoma de Barcelona, Barcelona, Spain; (7) Pulmonology Department, Hospital del Mar, and Universitat Pompeu Fabra, Parc de Recerca Biomèdica de Barcelona (PRBB), Barcelona, Spain; (8) Servei de Endocrinologia (Institut Clínic de Malalties Digestives i Metabòliques), Hospital Clínic, Barcelona, Spain.

**METHODS**

**Measurements**

The following measurements were obtained in all obese subjects before and 15±4 months after bariatric surgery (BS). Dyspnea was assessed using the modified Medical Research Council (mMRC) questionnaire [1]. The presence of metabolic syndrome (MS) was ascertained according to current recommendations [2]. Forced spirometry (before and after bronchodilation), plethysmographic lung volumes (Medisoft, Zoning de la Voie Cuivrée, Belgium) arterial blood gases (RapidPoint 500 systems, Siemens, Camberley, UK) and the 6-minute walking test (6MWT) were determined according to international recommendations [3-5]. Reference values were those of Roca *et al.* [6-8]. The presence of obstructive sleep apnea (OSAS) was investigated by polysomnography. An apnea/ hypopnea index (AHI) ≥15 events/h was considered indicative of OSAS [9].

Serum was obtained after overnight fasting by peripheral venopuncture followed by centrifugation and stored at -80ºC until analysis as previously reported [10]. The serum concentration of C-reactive protein (CRP) were determined using an immunoturbidimetry method (Advia Chemistry, Siemens Tarrytown, NY, USA) and those of leptin (Diagnostic Biochem Canada Inc. Ontario, Canada), serum adiponectin, soluble tumor necrosis factor-receptor 1(sTNF-R1), interleukin (IL)-8, IL-10 and 8-isoprostane by ELISA (US Biological Salem, MA, USA; IBL international Hamburg, Germany; ANOGEN Ontario, Canada and Cayman Chemical Company, Ann Arbor, MI, US, respectively). All biomarkers were quantified in duplicate and their mean values were used for analysis. In some individuals serum biomarker concentrations were below the lower limit of quantification (LLQ). To avoid a downward bias of biomarkers, a nominal level of half of the LLQ value was used in the analysis in individuals with values below the LLQ [11].

Exhaled breath condensate samples were obtained using an EcoScreen condenser (Jaeger, Würzburg, Germany) following international recommendations [12;13] and the concentrations of IL-8, IL-10 and 8-isoprostane were measured by ELISA (Cayman Chemical Company, Ann Arbor, MI, US).

**Statistical Analysis**

Results are described as mean ± standard deviation (SD), median [interquartile range] [IQR] or absolute and relative frequencies (%), as appropriate. Quantitative variables, were tested for normality using a Kolgomorov-Smirnov test and parametric (paired and unpaired t-test) and non-parametric (Wilcoxon and Mann-Whitney tests) were used accordingly to compare quantitative variables between patients and controls (at baseline) and between patients before and after BS. Fisher’s exact test and McNemar test were used for qualitative variables. Correlations between variables of interest were explored using the Spearman rank-correlation.

As previously reported [14], we used the 95^th^ (and 5^th^) percentile value determined in controls as the upper (and lower) normal levels, so biomarker concentrations beyond this threshold were considered abnormal in obese subjects. Cross tabulations between healthy and obese subjects, before and after BS, and also in different subsets of obese individuals according to sex, smoking status and coexistence of OSAS and MS, were determined to analyze biomarker alterations and their interactions. All statistical tests were two-sided and a p value <0.05 was considered significant. Due to the observational characteristics of this study p values presented were nominal and not adjusted for multiplicity. Data analysis was carried out with SPSS 20.0 (IBM Corporation).

Reference List

1. Mahler DA, Wells CK (1988) Evaluation of clinical methods for rating dyspnea. Chest; 93:580-586.

2. Alberti KG, Eckel RH, Grundy SM, Zimmet PZ, Cleeman JI et al. (2009) Harmonizing the metabolic syndrome: a joint interim statement of the International Diabetes Federation Task Force on Epidemiology and Prevention; National Heart, Lung, and Blood Institute; American Heart Association; World Heart Federation; International Atherosclerosis Society; and International Association for the Study of Obesity. Circulation; 120:1640-1645.

3. Miller MR, Hankinson J, Brusasco V, Burgos F, Casaburi R et al. (2005) Standardisation of spirometry. Eur Respir J; 26:319-338.

4. Wanger J, Clausen JL, Coates A, Pedersen OF, Brusasco V et al. (2005) Standardisation of the measurement of lung volumes. Eur Respir J; 26:511-522.

5. ATS Committee on Proficiency Standards for Clinical Pulmonary Function Laboratories (2002) ATS statement: guidelines for the six-minute walk test. Am J Respir Crit Care Med; 166:111-117.

6. Roca J, Burgos F, Sunyer J, Saez M, Chinn S et al. (1998) References values for forced spirometry. Group of the European Community Respiratory Health Survey. Eur Respir J; 11:1354-1362.

7. Roca J, Burgos F, Barbera JA, Sunyer J, Rodriguez-Roisin R et al. (1998) Prediction equations for plethysmographic lung volumes. Respir Med; 92:454-460.

8. Enright PL, Sherrill DL (1998) Reference equations for the six-minute walk in healthy adults. Am J Respir Crit Care Med; 158:1384-1387.

9. The Report of an American Academy of Sleep Medicine Task Force (Group of American Academy of Sleep Medicine, European Respiratory Society, Australasian Sleep Association and American Thoracic Society) (1999) Sleep-related breathing disorders in adults: recommendations for syndrome definition and measurement techniques in clinical research. Sleep; 22:667-689.

10. Rivas E, Arismendi E, Agusti A, Sanchez M, Delgado S et al. (2014) Pulmonary gas exchange abnormalities in obesity: findings one year after bariatric surgery. Chest [Under revision].

11. Muir K, Gomeni R (2004) Pharmacokinetics in Drug Development: Clinical Study Design and Analysis. Non-compartmental analysis. Bonate PL, Howard DR ed. pp. 235-266.

12. Horvath I, Hunt J, Barnes PJ, Alving K, Antczak A et al. (2005) Exhaled breath condensate: methodological recommendations and unresolved questions. Eur Respir J; 26:523-548.

13. Rodriguez-Trigo G, Zock JP, Pozo-Rodriguez F, Gomez FP, Monyarch G et al. (2010) Health changes in fishermen 2 years after clean-up of the Prestige oil spill. Ann Intern Med;153:489-498.

14. Agusti A, Edwards LD, Rennard SI, Macnee W, Tal-Singer R et al. (2012) Persistent systemic inflammation is associated with poor clinical outcomes in COPD: a novel phenotype. PLoS One; 7:e37483.

**FIGURE LEGENDS**

**Figure S1.** Systemic inflammome in obese participants classified according to sex before BS (for further explanation, see legend to Figure 2).

**Figure S2.** Systemic inflammome in obese participants classified according to smoking habits before BS. Current smokers (≥10 pack-years); non- (<10 pack-years) or former (>1 year after cessation) smokers (for further explanation, see legend to Figure 2).

**Figure S3.** Systemic inflammome in obese participants classified according to the presence or absence of obstructive sleep apnea syndrome (OSAS) before BS. OSAS was define as apnea/hypopnea index >15 events/hour (for further explanation, see legend of Figure 2).

**Figure S4.** Systemic inflammome in obese participants classified according to the presence or absence of metabolic syndrome (MS) before BS (for further explanation, see legend to Figure 2).
